# Evaluating the Effects of Laser Treatments on Visible Changes in the Photoaging Process of the Skin Using Specialized Measuring Devices

**DOI:** 10.3390/jcm13237439

**Published:** 2024-12-06

**Authors:** Aleksandra Podgórska, Aleksandra Kicman, Marta Wacewicz-Muczyńska, Tomasz Konończuk, Marek Niczyporuk

**Affiliations:** 1 Department of Aesthetic Medicine, Medical University of Bialystok, 15-267 Białystok, Poland; olakicman@gmail.com (A.K.); marek.niczyporuk@umb.edu.pl (M.N.); 2 Department of Specialist Cosmetology, Medical University of Bialystok, 15-267 Białystok, Poland; 3 Independent Researcher, 15-404 Białystok, Poland; kononczuktom@onet.eu

**Keywords:** laser, photoaging, ultrasonography, Courage & Khazaka, skin, treatment

## Abstract

**Background/Objectives**: The skin is the largest organ of the human body and is exposed to the aging process (not only chronological aging, but also photoaging). One of the methods used to correct visible skin changes due to photoaging is lasers. The aim of this study was to objectively evaluate the effect of Q-switched laser treatments on visible changes in the photoaging process among women using specialized measuring devices—ultrasound and Courage & Khazaka. **Methods**: The study included 36 women with visible signs of photoaged skin. The women were given a series of three treatments with a Fotona QX MAX fractional head laser. Both before and after the treatment, the women were examined for selected skin parameters with the help of specialized measuring devices such as Courage & Khazaka and skin ultrasound. Skin firmness and elasticity, the degree of hydration, TEWL and HL TOTAL levels, and MEP and HEP skin echogenicity were taken into account. The obtained results were tabulated and statistically analyzed. **Results**: Statistically significant differences were noted for parameters representing skin elasticity R2 [*p* = 0.0210] and R7 [*p* = 0.0302], TEWL [*p* = 0.0152] and HL TOTAL [*p* = 0.0367] on the forehead, and HL TOTAL [*p* = 0.0450] on the cheek. In addition, statistically significant differences were observed in the MEP/TP parameter on the forehead and cheek [*p* = 0.0236, 0.0475, respectively] and HEP/TP in the forehead area [*p* = 0.0367]. **Conclusions**: Q-switched laser treatments have a positive effect on the condition of women’s skin. Therapy with this laser reduces the visible changes in the photoaging process in the face.

## 1. Introduction

The skin is the largest biologically complex organ of the human body. It consists of three successive layers: the epidermis, dermis and subcutaneous tissue [1]. The epidermis is the layer of the skin that is in direct contact with the external environment, and it includes the stratum corneum, lamina propria, granular layer, squamous layer and basal layer [2]. The middle layer is the dermis, which is a connective tissue composed of fibroblasts and which is responsible for the biosynthesis of collagen, which gives the skin its elasticity and resistance to damage. In addition, it is involved in skin aging, wound healing and many other pathological processes [3,4,5,6]. The outermost layer of the skin is the subcutaneous tissue. It plays an important role in connecting the skin to muscle and bone tissue, is an energy store, and is also involved in skin repair processes after damage or infection [4,7]. The skin has many functions, including providing a suitable environment for deeper tissues by separating them from the external environment, as well as allowing the exchange of substances with the environment and receiving many stimuli from it. It provides protection against biological, chemical and physical agents. The skin is also an important sensory organ, and it plays an important role in regulating water and electrolyte balance and the immune response [2,8].

The skin also has a cosmetic effect. The process of organ aging occurs throughout a person’s life. Signs of skin aging become apparent with age, the degree of exposure to ultraviolet [UV] radiation or due to numerous chemical pollutants [4]. The aging process can be divided into chronological aging, which occurs naturally throughout the body and is caused by internal factors, and photoaging, which is caused by long-term exposure to UV radiation [4,9,10]. The process of photoaging involves not only the skin area, but also its supporting elements [11]. The place where the effects of photoaging appear depends largely on the wavelength of ultraviolet light. UV-A, with a wavelength of 320–400 nm, mainly affects the dermis, where it accelerates the hydrolysis of collagen fibers. In addition, it inhibits the production of hyaluronic acid [HA] [4,12,13]. UV-B [280–320 nm], on the other hand, affects keratinocytes, promoting mutations in their area, and potentially induces damage to DNA genetic material. In addition, aging fibroblasts present in the dermis cause hyperpigmentation and dark spots from the induction of the transcription process of the skin pigment gene melanin [4,14]. The main symptoms of photoaging are deep wrinkles, loss of skin elasticity, flabbiness, skin roughness and grayish-yellow color, dilated capillaries and numerous pigmentation spots [4,15]. The degree of the above-mentioned changes is influenced by skin type, geographic location and ethnicity, among other factors. Vulnerability to sunlight, on the other hand, depends on the melanin content of the skin. As such, according to the Fitzpatrick scale, skin types I, II and III are more sensitive to photoaging than IV, V or VI [16,17].

One of the techniques used for skin rejuvenation is lasers. The basis of laser beam action is the phenomenon of selective photothermolysis. According to this, the wavelength of the laser must be adequate to be absorbed by the target area with respect to healthy surrounding tissues, while the pulse duration must be shorter than the time required for heat loss by conduction [18,19,20,21].

Skin lesions associated with photoaging respond very well to Q-switched laser therapy [22]. We can divide these lasers by wavelength into Q-switched Ruby [694 nm], Q-switched Alexandrite [775 nm] and Q-switched Nd:YAG [1064 nm] [23]. Longer-wavelength laser beams are most often used to treat lesions in the dermis due to their greater penetration ability and lower absorption of the epidermis [21]. Consequently, Nd: YAG Q-switched lasers with a wavelength of 1064 nm are among those used to treat photoaging lesions. Because of their short pulse duration, they cause a photoacoustic effect in the tissue, rather than a photothermal effect like other lasers. The laser energy penetrates deep into the dermis, stimulating fibroblasts for the process of neocollagenesis and other proteins present in the skin [24]. The leveling of pigmented lesions present on the skin is accomplished by the above-mentioned process of selective thermolysis. Laser pulses reaching the target chromophore—the pigment—cause its degradation, additionally inducing, at the appropriate energy value, shock waves that stimulate the production of type III collagen fibers [21,25].

One method of imaging skin changes is skin ultrasonography [USG]. The functionality of ultrasound is based on the different reflection patterns in each tissue, through which it determines differences in the collagen, keratin or water content of the skin. In order to visualize superficial structures, its higher frequencies are used in dermatology [26,27]. As frequency increases, image resolution also improves, allowing finer details to be captured, increasing imaging precision [28]. This enables accurate identification of individual skin layers such as the epidermis, dermis and subcutaneous tissue [29].

The second method used to diagnose the condition of the skin is the Courage & Khazaka [C & K] modular device, which has specialized measuring heads for assessing various skin parameters, including skin hydration and lubrication, pH values, the degree of elasticity and firmness, and the level of transepidermal water loss from the epidermis [TEWL] [30].

Therefore, the purpose of the present study was to objectively evaluate the effect of Q-switched laser treatments on visible changes in the photoaging process among women using specialized measuring devices—ultrasound and Courage & Khazaka.

## 2. Materials and Methods

### 2.1. Patients

The study group consisted of 36 women between the ages of 40 and 75. The average age of the respondents was 48 years [Table 1]. The women who participated in the study were those with visible signs of the skin photoaging process, which are pigmentation changes, wrinkles, dry and sagging skin, and dilated blood vessels. The women were exclusively Caucasian, and thus of skin phototype II or III according to the Fitzpatric scale. Each of the women was acquainted with the details of the study and gave written consent before starting the series of treatments. The study was approved by the Bioethics Committee of the Medical University of Bialystok with the following number: APK.002.428.2021.

### 2.2. Q-Switched Laser Therapy

Patients underwent laser therapy using a Fotona QX MAX laser. It is a Q-switched laser based on Nd:Yag crystal with a wavelength reaching 1064 nm and a pulse length equal to 5 ns. In addition, the FS 20A fractional head was used for the treatments. The therapy consisted of 3 treatments performed at 4–6-week intervals. Each patient was given 2 overlapping crosswalks over the treated area during one session using 1 J/cm^2^ energy and at a pulse frequency of 4 Hz. The applied treatment power of 1 J/cm^2^ is the dose indicated in the recommended treatment protocols; hence, this radiation dose was applied to all study patients. Given the skin phototype represented by the entire study group, the radiation dose did not require adjustment. Prior to the series of treatments, each woman underwent skin examinations using specialized measuring devices, which were ultrasonography and the Courage & Khazaka modular device, where heads were used to measure skin firmness and elasticity, skin hydration and TEWL assessment.

### 2.3. Skin Assessment

Women taking part in the study were asked to report for the tests without applying facial skin care or makeup in order to obtain reliable results. Measurements were taken in two areas of the face—the forehead and cheek. The patients rested for about 20 min before taking the measurements. Measurements were taken at a temperature of about 20 degrees Celsius and a humidity level of 40–60%.

#### 2.3.1. Assessment of Skin Firmness and Elasticity, Skin Surface Hydration and Transepidermal Water Loss

A scientific Cutometer^®^ dual MPA 580 multi-probe system [Courage & Khazaka Electronic, Köln, Germany] was used to assess the skin condition of the women under study. It is a device consisting of a basic device, the so-called base, and a set of measuring probes that allow for professional measurements not only of skin parameters, but also of hair. The system provides the possibility of connecting multiple probes, including those for measuring skin surface hydration [Corneometer^®^ CM 825 (Courage & Khazaka Electronic, Köln, Germany)], skin viscoelasticity [Cutometer^®^], transepidermal water loss from the epidermis [Tewameter^®^ TM Hex], pH measurement [Skin pH-Meter PH 905] or the degree of skin oiliness [Sebumeter^®^ SM 815] [31].

In the present study, 3 probes were used to assess the condition of women’s skin: the Cutometer^®^, Corneometer^®^ CM 825 and Tewameter^®^ TM Hex.

The Cutometer^®^ is used to measure the elasticity of the upper layers of the skin using a vacuum, causing mechanical deformation of the skin. The principle of the probe is based on the suction method. The pressure created inside the device draws the skin into the small opening of the probe, where the depth of penetration is determined using a non-contact optical measuring system, and then the skin is released after a given time. The skin’s resistance to the vacuum—firmness—acting inside the probe, as well as its ability to return to its original state—elasticity—are displayed as curves in the actual measurement time. From these, many parameters related to elasticity and viscoelasticity of the skin surface and skin aging can be calculated [30,31,32]. In the current study, parameters such as R0, R1, R2, R5 and R7 were evaluated.

The Corneometer^®^ CM 825 is used to measure the hydration of the surface of the skin, mainly its top horny layer. The device measures the capacitance of the dielectric medium of the stratum corneum, whose dielectric properties change as the degree of hydration increases. The measurement is based on differences in the dielectric constant of water, which is 81, and other substances with a constant of mostly less than 7. Located at the top of the sensor in the probe head, the paths, which are separated by a glass plate from the skin surface, produce an electric field between them due to the interaction of potentials of opposite signs. One of the paths has an excess of electrons [minus], while the other is deficient [plus]. Once the probe is placed on the skin surface, the scattering field penetrates the superficial layer of the skin. The device measures changes in the dielectric constant due to skin hydration, which is altered by the capacitance of a precision capacitor. This measurement is capable of detecting the smallest changes that have occurred in the degree of hydration of the skin surface [31,33].

The Tewameter^®^ TM Hex is used to measure transepidermal water loss [TEWL] and is an important parameter for assessing skin barrier function. Weakening of this barrier is reflected by high TEWL values and is associated with increased epidermal permeability. The device uses the “open chamber” technique, which is the only continuous measurement method. Moreover, it does not disturb the skin microenvironment. Two sensors are present inside the probe, both for measuring relative humidity and temperature. It measures the gradient of water evaporation density from the skin surface indirectly, which is proportional to the TEWL value [34,35]. The current study evaluated the TEWL and total heat loss [HL TOTAL] parameters.

#### 2.3.2. Assessment of Skin Echogenicity

The second measurement method was skin ultrasonography. An ultrasound machine, DermaMed [Dramiński S.A., Olsztyn, Poland], was used for the study, consisting of three main components: a head [probe] with an ultrasound transducer, specialized software and a user panel. The frequency of the transducer was 48 MHz, while the scanning depth reached 30 mm [36]. The device presented the image in real time, in B-mode. This is a type of image presentation that allows a set of points to be displayed as a two-dimensional image. The echo returning to the transducer is converted into points whose width corresponds to the echo at its base, while the brightness corresponds to the amount of energy received. Consequently, the greater the amount of waves reflected, the brighter the points. The amount of energy returned is a function of time encoded on a color scale of 0 to 255. Black areas, regardless of the encoding method, are referred to as anechoic, while white areas are referred to as hyperechoic [26,37]. The evaluation of echogenicity is very important in the measurement of aging skin, as it usually shows an increase when correlated with an increase in collagen production and a decrease in the amount of intercellular matrix. An objective assessment of echogenicity can be made based on the number and average intensity of pixels. We divided it into three categories on this basis. The first is low-echogenicity pixels [LEPs], covering a range of 0 to 30; the second category is composed of medium-echogenicity pixels [MEPs], with a range of 50 to 150; and the last category is high-echogenicity pixels [HEPs], with a range of 200 to 255. LEPs quantify the degree of skin hydration, solar elastosis, collagen fiber degradation and inflammatory processes occurring in the skin. MEPs and HEPs, on the other hand, measure the structure of collagen, elastin and microfibrils [27,38,39,40]. In the present study, MEP and HEP counts were examined.

### 2.4. Statistical Analysis

The results obtained were analyzed using the Statistica V 13.3. statistical program and Microsoft Office Excel 2019 software. The Shapiro–Wilk W test was used to assess the normality of the data distribution. A non-parametric distribution of the data was shown. Parameters of descriptive statistics, i.e., mean, median, minimum, maximum, interquartile range and standard deviation, were also calculated. Differences between dependent groups were tested with the non-parametric Mann–Whitney U test. The level of statistical significance was taken as *p* < 0.05.

**Figure 1:**
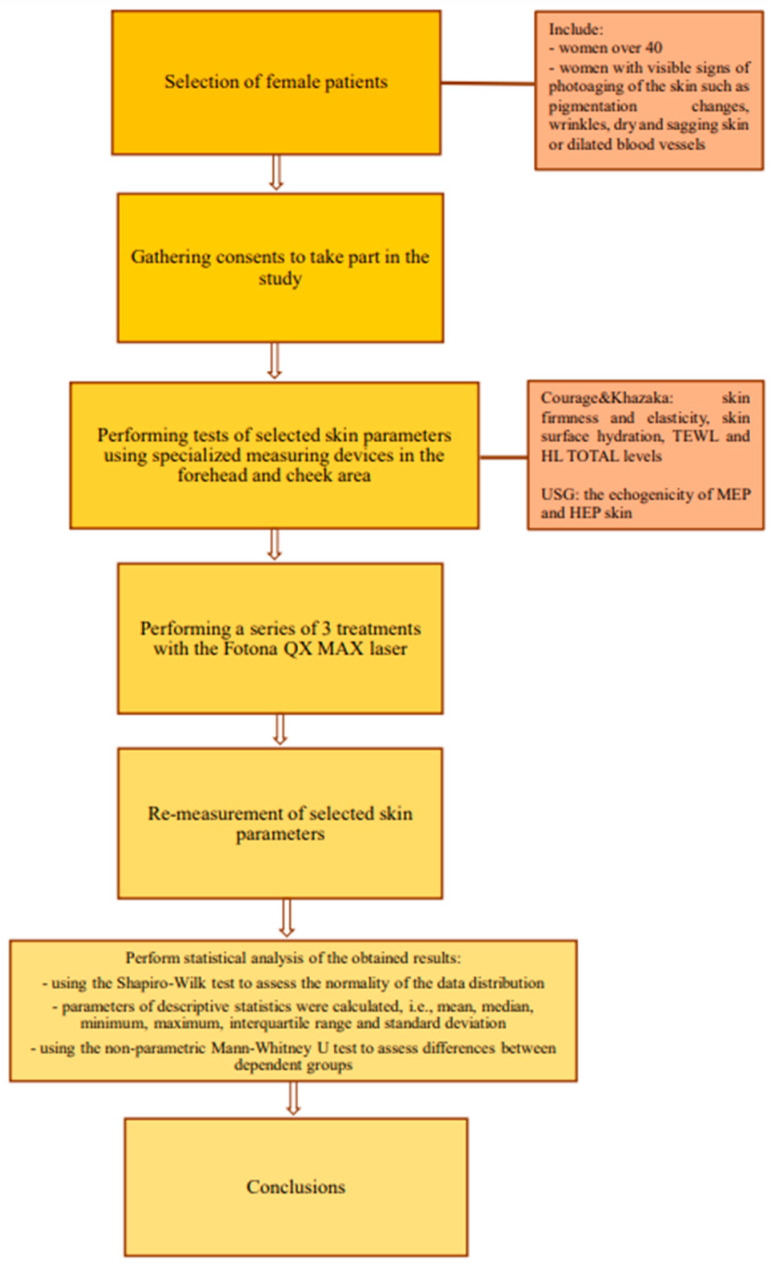
Flowchart of the study course.

The entire course of the scientific experiment is shown as a flowchart [Figure 1].

## 3. Results

### 3.1. Characterization of the Changes Occurring in the Parameters of Skin Firmness and Elasticity, Skin Surface Hydration, and TEWL and HL TOTAL

Changes in all descriptive statistics’ values for the skin parameters studied with the Courage + Khazaka device, both before and after the series of laser treatments, are shown in Table 2.

#### 3.1.1. Skin Firmness [R0 and R1]

The value of the mean of the first parameter studied, which was the R0 parameter, which is responsible for the level of skin firmness, decreased after the series of treatments, indicating that the treatment had a positive effect on skin firmness. These changes occurred both in the forehead area, where the average before was 0.1828, while after it was equal to 0.1696, and in the cheek, where the values were 0.2694 and 0.2486, respectively. The lower the R0 parameter values, the firmer the skin.

The average of the second parameter, indicating the level of firmness of the skin, R1, similarly to the above R0 parameter, decreased after the series of treatments. Before the therapy, it had values equal to 0.0649 for the forehead and 0.0882 for the cheek, and after the therapy these were 0.0518 and 0.0770, respectively. For the value of R1, identically to R0, lower values show improved firmness of the skin.

#### 3.1.2. Skin Elasticity [R2, R5 and R7]

The third parameter is R2, whose higher mean value indicates improved skin elasticity. The mean, in both the forehead and cheek areas, increased after the treatments, so the skin became more elastic. Its value before the treatments was 72.93 on the forehead and 66.30 on the cheek, while after it adopted values of 79.68 and 69.54, respectively.

The R5 parameter shows an identical relationship to R2. The higher its value, the better the elasticity of the skin. In the forehead area, the average of the R5 parameter before the series of treatments was 64.41, while after it was equal to 70.39, and on the cheek the values were equal to 55.87 and 59.72, respectively.

The last of the studied parameters, which speaks of the degree of skin elasticity, is the R7 parameter, the average of which increased in both study areas. On the forehead, it was 40.92 before therapy and on the cheek it was 38.35, while after therapy it had values equal to 46.76 and 42.17. This shows the positive effect of laser treatments on skin elasticity.

#### 3.1.3. Hydration of the Skin Surface

The degree of hydration of the skin surface in the forehead area increased from a mean of 56.00 to 61.82. A similar improvement in hydration was observed in the other area, where the mean before the series of treatments was 40.88, while after the value increased to 45.38.

#### 3.1.4. Water and Heat Loss from the Epidermis

TEWL and HL TOTAL decreased, indicating that the laser treatments had a positive effect on these parameters. The mean value of TEWL on the forehead before and after the treatments was 15.13 and 12.96, respectively, while on the cheek it was 12.84 and 11.40, respectively. The mean value of the HL TOTAL parameter in the forehead area, on the other hand, was 14.12 before therapy and 12.32 after, while on the cheek it was 17.82 before and 15.94 after.

### 3.2. Characteristics of Changes in Skin Echogenicity

Changes in skin echogenicity described using descriptive statistics are shown in Table 3.

#### 3.2.1. Medium-Frequency Pixels/Total Pixels

The percentage of pixels with an average MEP frequency relative to the total number of TP pixels decreased after the treatments in both the forehead and cheek areas. The average MEP/TP was 39.36 on the forehead before treatment and decreased to a value of 28.88 after treatment. On the cheek, the values were 51.30 and 39.05, respectively. The lower the percentage of MEPs, the better.

#### 3.2.2. High-Frequency Pixels/Total Pixels

The percentage of high-frequency HEPs to total pixels (TPs) increased in contrast to MEPs, which indicates the positive effect of the treatments on skin condition. The higher the HEP value, the better. Before therapy, the average HEPs/TPs in the forehead area was 61.52, and after it was equal to 71.44; on the cheeks, the averages were 47.94 and 60.71, respectively.

### 3.3. Assessment of Differences Between Groups with the Help of the Mann–Whitney U Test

Despite the obtained changes in the mean values of all tested skin parameters, both with the Courage & Khazaka device and ultrasound, in both facial areas, statistical significance was not found in all these parameters. The data are shown in Table 4 and Table 5.

#### 3.3.1. Courage & Khazaka Parameters

**Figure 2:**
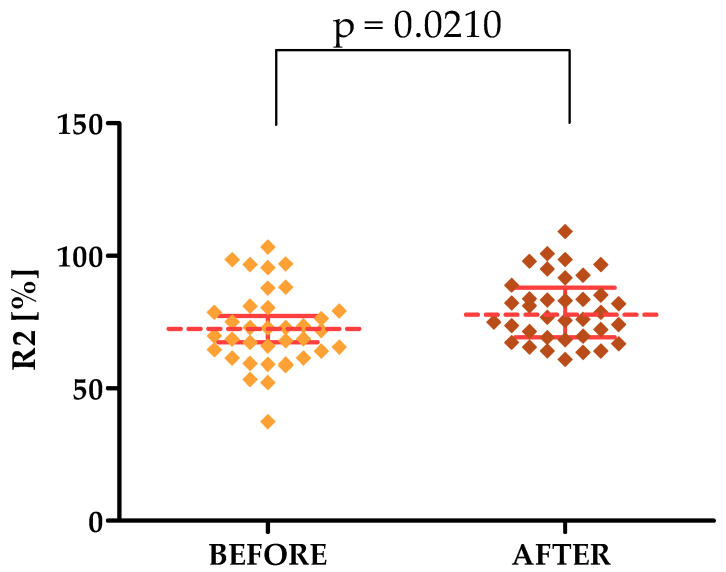
Changes in the R2 parameter after a series of treatments in the forehead area.

**Figure 3:**
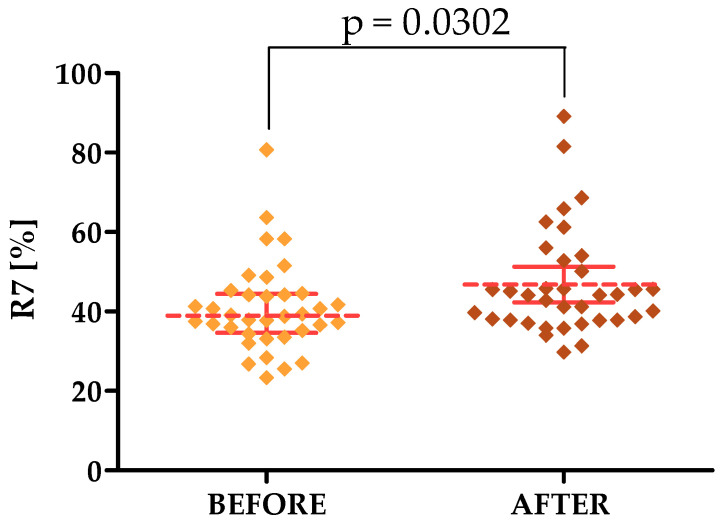
Changes in the R7 parameter after a series of treatments in the forehead area.

**Figure 4:**
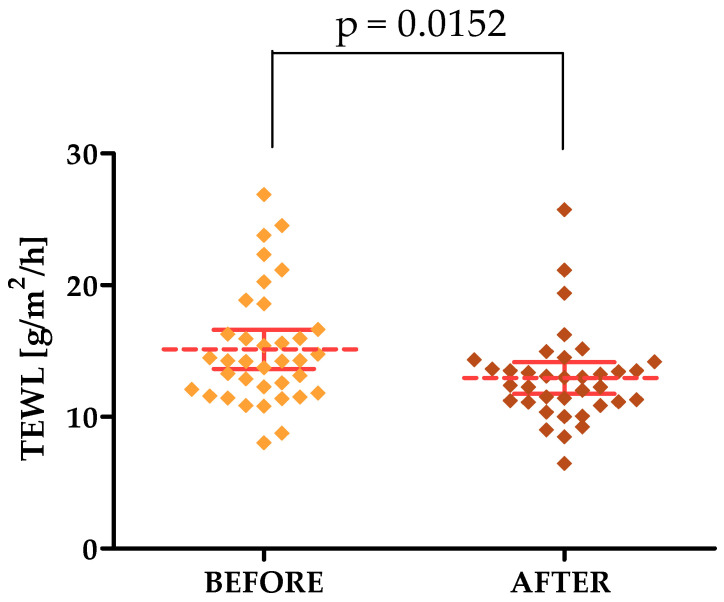
TEWL changes after a series of treatments in the forehead area.

**Figure 5:**
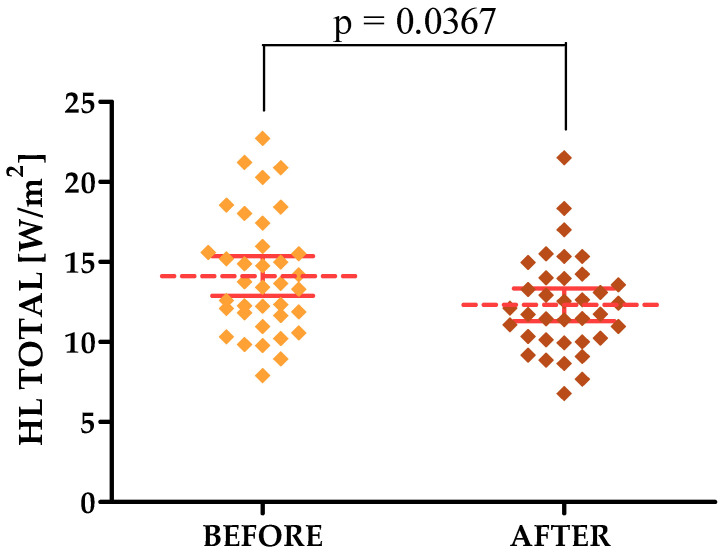
HL/TOTAL changes after a series of treatments in the forehead area.

**Figure 6:**
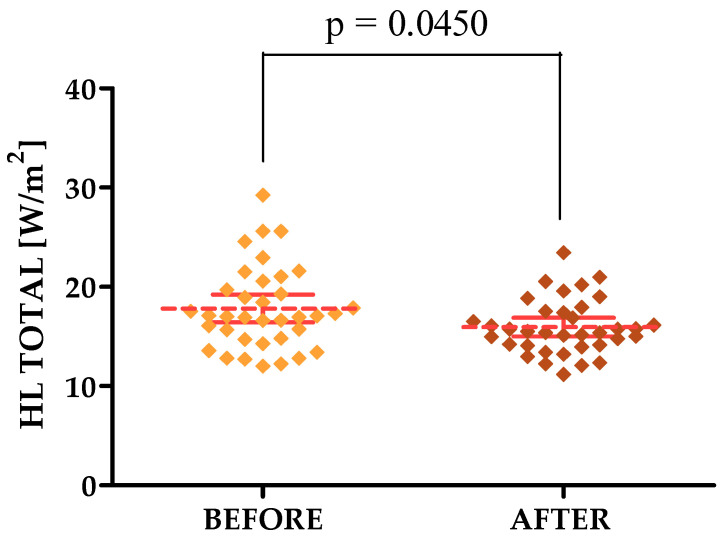
HL/TOTAL changes after a series of treatments in the cheek area.

For the parameters tested with the Courage & Khazaka modular device, statistically significant differences were shown in the forehead area for both the R2, R7, TEWL and HL TOTAL parameters, for which differences were observed for the cheek area. The *p*-values were 0.0210, 0.0302, 0.0152, 0.0367 and 0.0450, respectively [Figure 2, Figure 3, Figure 4, Figure 5 and Figure 6].

#### 3.3.2. Ultrasound Parameters

**Figure 7:**
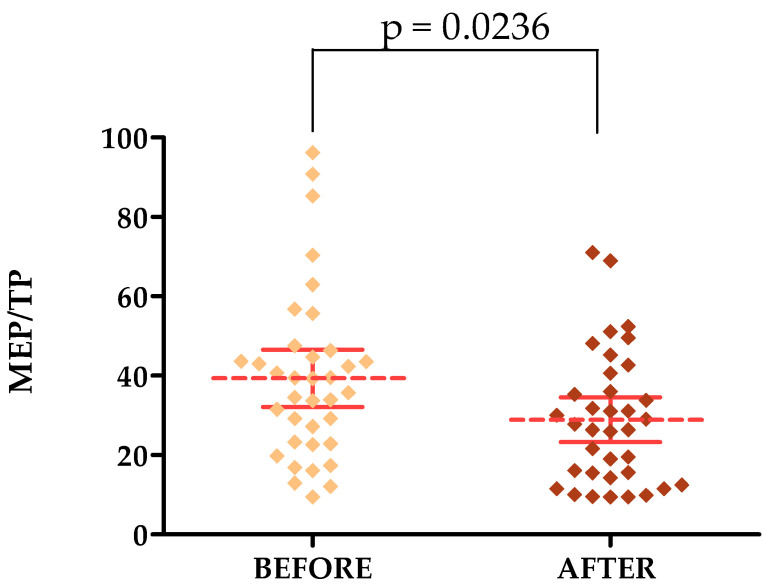
MEP/TP changes after a series of treatments in the forehead area.

**Figure 8:**
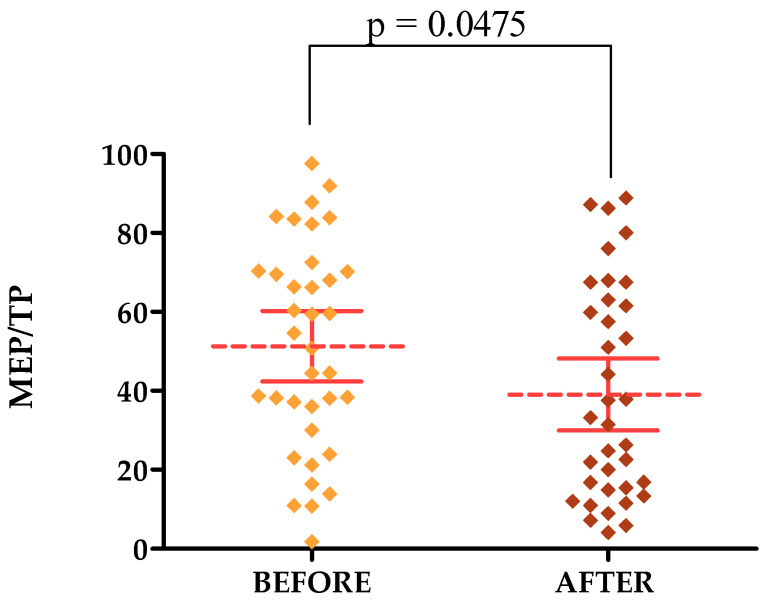
MEP/TP changes after a series of treatments in the cheek area.

**Figure 9:**
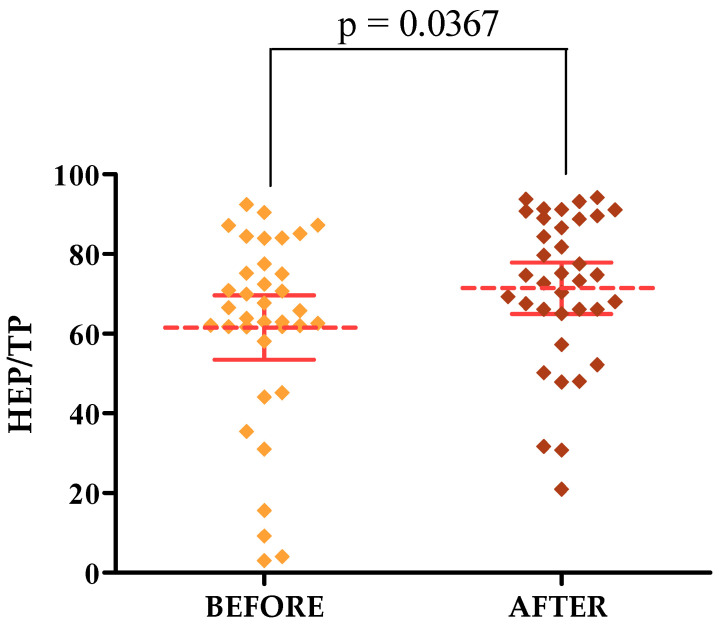
HEP/TP changes after a series of treatments in the forehead area.

On skin ultrasound, statistically significant differences were observed for MEP/TP parameters in the forehead and cheek area and HEPs/TPs in the forehead area only. HEPs/TPs in the cheek area showed no statistically significant difference. The *p*-value for MEPs/TPs on the forehead was 0.0236 [Figure 7] and on the cheek it was 0.0475 [Figure 8], while the *p*-value for HEPs/TPs on the forehead was 0.0367 [Figure 9].

## 4. Discussion

The skin is the largest human organ; in addition to its numerous physiological functions, it also has an important cosmetic function. The condition of the skin has a significant impact on people’s well-being. Patients with dermatological problems have lower levels of self-esteem and the condition of the skin has a significant impact on their psychological and social state [41,42]. One of the most significant processes associated with skin deterioration is photoaging, which is dependent on ultraviolet light [12,13]. The condition of the skin can be improved by various methods, which include specialized care performed by skilled medical personnel. These include laser therapies; Q-switched laser therapy has been shown to have particular potential, and is associated with neocollagenesis processes and the breakdown of unwanted pigment [21,25].

Also important in the context of skin care is the proper diagnosis of potential changes occurring within the skin. Currently, one of the best diagnostic cosmetological tools is ultrasound, which is a non-invasive method, and the Courage & Khazaka modular device, which allows for the evaluation of specialized skin parameters such as hydration and lubrication, pH values, the degree of elasticity and firmness, and the level of transepidermal water loss from the epidermis [28,29,30].

Therefore, the purpose of this study was to evaluate the effects of Q-switched laser treatments on skin changes associated with photoaging using non-invasive and specialized diagnostic methods—ultrasound and Courage & Khazaka.

In our study, we considered several key skin parameters such as skin firmness, elasticity and hydration. After a series of Q-switched laser treatments, we found an improvement in skin firmness in the forehead and cheeks expressed through the R0 parameter. We are now the first research team to determine the effect of Q-switched laser treatments on this skin parameter, so we will relate some of our results to work partially similar to ours. R0 is currently an under-reported parameter within dermatology and cosmetology. A single study conducted by Delavie Science indicates that following treatment with an anti-aging serum for 28 days was also associated with an improvement in skin firmness expressed by R0 [43]. This also confirms the results obtained by our research team and indicates that the determination of the R0 parameter value was performed correctly and that the parameter itself can be used in future studies to determine facial skin firmness. Importantly, the team of Brancalion Catapani et al., 2016 using the Courage & Khazaka measuring device on the skin of the forearm also indicated an improvement in skin firmness after treatments with therapeutic ultrasound, expressed by the R0 parameter [44]. The R1 parameter also indicates the level of skin firmness. In our study, we showed a significant decrease in the value of this index, which indicates an improvement in skin firmness. The R1 and R0 parameters are related to each other; the decrease in the value of R1 and R0 after the treatments clearly indicates the positive effect of Q-switched laser therapy on skin firmness and the correct methodology of our study. It is unfortunate that the R1 parameter is currently not very common in dermatology, so we are unable to relate our results to the studies of other research teams. A single study by Oh et al., 2018 indicates the possibility of using the R1 parameter to assess skin firmness around the eyes [45].

Another parameter studied in our experiments is elasticity as expressed by the R2 parameter; in our study, the R2 value on both the forehead and cheeks increased, indicating improved skin elasticity after Q-switched laser treatments. Significantly, a single study by Kolodziejczak and Rotsztejn [2022] determining the effect of non-ablative fractional lasers on skin elasticity showed no effect of treatments on R2 value [46]. This may indicate the superiority of Q-switched laser treatments and the potential of their use exclusively within the skin of the cheeks and forehead. This is partially confirmed by the study of Shin et al., 2011, who found an increase in the R2 parameter value within the facial skin after performing treatments with intense pulsed light [47]. Closely related to the R2 parameter is the R5 parameter which also indicates the level of skin elasticity. The results we obtained show an increase in R5 values after the cosmetic treatments performed. This also partially agrees with the study of Shin et al., 2011, who found an increase in R5 values after performing a series of treatments with intense pulsed light. The last of the parameters we determined related to skin elasticity is R7; again, we found an increase in the value of this parameter after laser treatments. Our study partially agrees with the study of Kolodziejczak and Rotsztejn [2017], who also found an increase in R7 in the skin around the eyes after cosmetic treatments—an ablative fractional laser, non-ablative radiofrequency and an intense light source [48]. It should also be noted that performing simultaneous measurements of R2, R5 and R7 better reflects the changes in the level of skin elasticity than performing tests of single parameters [48], which indicates the validity of their selection in our study.

We found a statistically significant improvement in skin hydration after Q-switched laser treatments. Adequate skin hydration translates into proper skin function and disorders in skin hydration are associated with the risk of developing dermatological diseases [49]. Our study indicates not only the potential of Q-switched laser treatments in improving skin hydration, but also the possibility of using ultrasound and Courage & Khazaka instrumentation in assessing water loss. Indeed, the assessment of skin hydration levels is essential in dermatological and cosmetic studies [49].

Transepidermal water loss depends on many variables, such as age and anatomical location. Namely, water loss is associated with the possibility of developing certain dermatological diseases [50]. In our study, we showed that Q-switched laser treatments have a positive effect on the decrease in epidermal water loss. In addition, our research indicates that the Tewameter^®^ is a good tool for assessing TEWL, and this is also confirmed by studies conducted by other research teams [51].

The last parameter we studied was the effect of Q-switched laser treatments on the structure of collagen, elastin and microfibrils expressed by evaluating three parameters—low-echogenicity pixels [LEPs], medium-echogenicity pixels [MEPs] and high-echogenicity pixels [HEPs]. The results we obtained indicate that Q-switched laser treatments have a positive effect on the parameters studied and thus on the structure of the most important skin proteins. Collagen, elastin and microfibrils are the most important structural proteins within the skin. Proper collagen content translates into skin elasticity; elastin is responsible for skin’s stretch, while microfibrils are responsible for, among other things, maintaining skin homeostasis and imparting biomechanical properties to the skin. A decrease in the content of these proteins in the skin translates into a deterioration of its properties and translates into a visual deterioration of the skin [52,53,54]. The use of Q-switched laser treatments can increase the content of these proteins in the skin and thus translate into improved skin properties.

However, our study has some limitations. The first is the number of female patients participating in the study. Since our study was a pilot study, a limited number of female patients participated in the study. In the study’s next steps, we plan to include a larger number of female patients. The second limitation was the age of the patients, as all the women were over 40 years old; in future studies, we also want to include younger patients. The last limitation is the gender of the patients—in our study, there were only women. In future studies, we also want to evaluate the effect of Q-switched laser treatments on the skin parameters of men—this will allow us to determine the potential impact of sex hormones.

In conclusion, our study showed a positive effect of Q-switched laser treatments on skin parameters such as elasticity, resilience, epidermal water loss and the composition of key structural proteins within the skin. This indicates the potential for the wider use of this type of laser treatment for improving skin quality. In addition, we demonstrated the potential of the Courage & Khazaka measuring device in assessing physiological parameters of the skin.

## 5. Conclusions

Q-switched laser treatments have a positive effect on the condition of women’s skin. Therapy with this laser reduces visible changes in the photoaging process on the face, and the post-treatment recovery process is shorter than that with lasers that induce a photothermal effect.

## Figures and Tables

**Table 1:** Characteristics of the study group [n = 36].

Statistical Parameters	AGE [YEARS]
Av. ± SD	48 ± 9
Min–Max	40–75
Med.	46
IQR	41–53

Av.—average; IQR—interquartile range; Max—maximum; Med.—median; Min—minimum; SD—standard deviation.

**Table 2:** Descriptive characteristics of selected skin parameters before and after laser therapy using a specialized Courage-Khazaka measuring device.

C&K PARAMETERS	BEFORE				AFTER			
	Av. ± SD	Min–Max	Med.	IQR	Av. ± SD	Min–Max	Med.	IQR
**R0 [mm] [FOREHEAD]**	0.1828 ± 0.0823	0.0380–0.4050	0.1805	0.1135–0.2353	0.1696 ± 0.0709	0.0410–0.3450	0.1705	0.1165–0.2138
**R0 [mm] [CHEEK]**	0.2694 ± 0.1020	0.1070–0.4390	0.2860	0.1735–0.3605	0.2486 ± 0.0915	0.0830–0.4270	0.2525	0.1730–0.3210
**R1 [mm] [FOREHEAD]**	0.0649 ± 0.0384	0.0160–0.1880	0.0575	0.0363–0.0740	0.0518 ± 0.0246	0.0150–0.1340	0.0535	0.0320–0.0668
**R1 [mm] [CHEEK]**	0.0882 ± 0.0320	0.0280–0.1480	0.0900	0.0613–0.1150	0.0770 ± 0.0321	0.0200–0.1340	0.0770	0.0543–0.1025
**R2 [%] [FOREHEAD]**	72.93 ± 14.50	37.40–103.30	70.80	62.08–80.10	79.68 ± 12.24	60.90–109.20	77.75	69.33–97.98
**R2 [%] [CHEEK]**	66.30 ± 10.79	39.20–93.50	64.50	60.30–73.73	69.54 ± 10.59	48.20–93.80	68.30	63.10–15.55
**R5 [%] [FOREHEAD]**	64.41 ± 16.28	27.40–93.70	64.50	56.83–77.45	70.39 ± 13.63	49.80–115.90	66.85	60.40–79.30
**R5 [%] [CHEEK]**	55.87 ± 12.72	36.70–90.90	55.25	45.50–64.88	59.72 ± 13.49	43.10–97.30	57.85	49.70–64.08
**R7 [%] [FOREHEAD]**	40.92 ± 11.31	23.40–80.70	38.95	34.60–44.43	46.76 ± 13.29	29.80–89.10	44.10	37.90–52.13
**R7 [%] [CHEEK]**	38.35 ± 8.93	24.30–58.40	35.85	30.55–44.15	42.17 ± 9.66	28.60–68.00	39.35	35.63–47.33
**CM [FOREHEAD]**	56.00 ± 14.07	27.77–80.80	56.63	45.00–66.82	61.82 ± 12.45	25.73–78.98	64.24	53.93–71.68
**CM** **[CHEEK]**	40.88 ± 12.96	18.73–74.77	39.08	31.33–50.90	45.38 ± 13.96	21.70–72.93	45.95	34.33–53.95
**TEWL [g/m^2^/h] [FOREHEAD]**	15.13 ± 4.41	8.04–26.89	14.25	11.87–16.54	12.96 ± 3.54	6.47–25.74	12.69	11.11–14.06
**TEWL [g/m^2^/h] [CHEEK]**	12.84 ± 3.85	7.54–23.76	12.74	10.16–14.14	11.40 ± 2.85	6.39–17.91	10.98	9.44–12.86
**HL TOTAL [W/m^2^] [FOREHEAD]**	14.12 ± 3.67	7.90–22.71	13.54	11.69–15.88	12.32 ± 3.02	6.78–21.50	11.93	10.16–14.00
**HL TOTAL [W/m^2^] [CHEEK]**	17.82 ± 4.16	12.03–29.26	17.06	14.75–20.37	15.94 ± 2.77	11.20–23.45	15.47	14.09–17.53

Av.—average; IQR—interquartile range; Max—maximum; Med.—median; Min—minimum; SD—standard deviation; TEWL—transepidermal water loss; HL TOTAL—heat loss total.

**Table 3:** Descriptive characteristics of skin echogenicity before and after laser treatments using ultrasound.

USG PARAMETERS	BEFORE				AFTER			
	Av. ± SD	Min–Max	Med.	IQR	Av. ± SD	Min–Max	Med.	IQR
**MEP/TP [FOREHEAD]**	39.36 ± 21.28	9.42–96.20	37.49	23.01–45.92	28.88 ± 16.62	9.42–71.04	27.09	14.61–39.46
**MEP/TP [CHEEK]**	51.30 ± 26.28	1.75–97.59	52.70	31.55–70.36	39.05 ± 26.96	4.11–88.84	32.32	15.00–62.67
**HEP/TP [FOREHEAD]**	61.52 ± 23.93	3.06–92.46	64.81	59.05–76.96	71.44 ± 19.05	20.98–94.23	74.00	65.30–88.99
**HEP/TP [CHEEK]**	47.94 ± 27.76	2.84–97.60	45.65	20.67–71.67	60.71 ± 27.75	2.81–96.45	71.51	36.58–85.39

Av.—average; HEPs—high-echogenicity pixels; IQR—interquartile range; Max—maximum; Med.—median; MEPs—medium-echogenicity pixels; Min—minimum; SD—standard deviation; TPs—total pixels.

**Table 4:** Differences in the degree of skin firmness and elasticity, skin hydration, TEWL and HL TOTAL levels among the women studied.

C & K PARAMETERS	AV. BEFORE	AV. AFTER	*p*-VALUE
**R0 [mm] [FOREHEAD]**	0.1828	0.1696	0.5733
**R0 [mm] [CHEEK]**	0.2694	0.2486	0.3189
**R1 [mm] [FOREHEAD]**	0.0649	0.0518	0.1641
**R1 [mm] [CHEEK]**	0.0882	0.0770	0.1765
**R2 [%] [FOREHEAD]**	72.93	79.68	0.0210 *
**R2 [%] [CHEEK]**	66.30	69.54	0.2217
**R5 [%] [FOREHEAD]**	64.41	70.39	0.1510
**R5 [%] [CHEEK]**	55.87	59.72	0.2370
**R7 [%] [FOREHEAD]**	40.92	46.76	0.0302 *
**R7 [%] [CHEEK]**	38.35	42.17	0.0631
**CM [FOREHEAD]**	56.00	61.82	0.0507
**CM [CHEEK]**	40.88	45.38	0.1748
**TEWL [g/m^2^/h] [FOREHEAD]**	15.13	12.96	0.0152 *
**TEWL [g/m^2^/h] [CHEEK]**	12.84	11.40	0.0901
**HL TOTAL [W/m^2^] [FOREHEAD]**	14.12	12.32	0.0367 *
**HL TOTAL [W/m^2^] [CHEEK]**	17.82	15.94	0.0450 *

*—statistically significant value; Av.—average; TEWL—transepidermal water loss; HL TOTAL—heat loss total.

**Table 5:** Differences in the echogenicity of the skin of the women studied.

USG PARAMETERS	AV. BEFORE	AV. AFTER	p-VALUE
**MEP/TP [FOREHEAD]**	39.36	28.88	0.0236 *
**MEP/TP [CHEEK]**	51.30	39.05	0.0475 *
**HEP/TP [FOREHEAD]**	61.52	71.44	0.0367 *
**HEP/TP [CHEEK]**	47.94	60.71	0.0534

*—statistically significant value; Av.—average; HEPs—high-echogenicity pixels; IQR—interquartile range; Max—maximum; Med.—median; MEPs—medium-echogenicity pixels; Min—minimum; SD—standard deviation; TPs—total pixels.

## Data Availability

The original contributions presented in the study are included in the article, further inquiries can be directed to the corresponding author/s.
